# Human STING is a proton channel

**DOI:** 10.1126/science.adf8974

**Published:** 2023-08-03

**Authors:** Bingxu Liu, Rebecca J. Carlson, Ivan S. Pires, Matteo Gentili, Ellie Feng, Quentin Hellier, Marc A. Schwartz, Paul C. Blainey, Darrell J. Irvine, Nir Hacohen

**Affiliations:** 1 Broad Institute, Cambridge, MA, USA.; 2 Department of Biology, Massachusetts Institute of Technology, Cambridge, MA, USA.; 3 The Koch Institute for Integrative Cancer Research at MIT, Cambridge, MA, USA.; 4 Massachusetts Institute of Technology, Department of Health Sciences and Technology, Cambridge, MA, USA.; 5 Massachusetts Institute of Technology, Department of Biological Engineering, Cambridge, MA, USA.; 6 Department of Pediatrics, Harvard Medical School, Boston, MA, USA.; 7 Division of Hematology and Oncology, Boston Children’s Hospital, Boston, MA, USA.; 8 Department of Pediatric Oncology, Dana-Farber Cancer Institute, Boston, MA, USA.; 9 Massachusetts General Hospital Cancer Center, Boston, MA, USA.

## Abstract

Proton leakage from organelles is a common signal for noncanonical light chain 3B (LC3B) lipidation and inflammasome activation, processes induced upon stimulator of interferon genes (STING) activation. On the basis of structural analysis, we hypothesized that human STING is a proton channel. Indeed, we found that STING activation induced a pH increase in the Golgi and that STING reconstituted in liposomes enabled transmembrane proton transport. Compound 53 (C53), a STING agonist that binds the putative channel interface, blocked STING-induced proton flux in the Golgi and in liposomes. STING-induced LC3B lipidation and inflammasome activation were also inhibited by C53, suggesting that STING’s channel activity is critical for these two processes. Thus, STING’s interferon-induction function can be decoupled from its roles in LC3B lipidation and inflammasome activation.

**S**timulator of interferon genes (STING) is a conserved mammalian cytoplasmic receptor that is essential for sensing cyclic dinucleotides derived directly from bacteria (1) or synthesized by cyclic GMP-AMP (cGAMP) synthase (cGAS) upon recognition of cytosolic DNA (2, 3). Upon binding to its native ligand, cGAMP, STING undergoes a conformational change and translocates from the endoplasmic reticulum (ER) to the Golgi and endosomes, where it carries out multiple biological functions, including interferon induction (4), noncanonical light-chain 3B (LC3B) lipidation (5), and NOD-like receptor family pyrin domain–containing 3 (NLRP3) inflammasome activation (6). Whereas interferon is induced by STING-mediated activation of TANK-binding kinase 1 (TBK1) and interferon regulatory factor 3 (IRF3) (7, 8), the mechanisms by which STING activates noninterferon functions, in particular noncanonical LC3B lipidation and inflammasome activation, are still unclear.

STING induces focal adhesion kinase family interacting protein of 200 kDa (FIP200)–independent noncanonical LC3B lipidation, which involves conjugation of autophagy-related protein 8 (ATG8) to single membranes (CASM) (9, 10). This process, sometimes termed “noncanonical autophagy” (11), is important for bacterial control (12) and is known to be initiated by ion release into the cytoplasm from acidic organelles (such as Golgi and endosomes) through multiple mechanisms, including organelle membrane damage (12), pathogen-derived ion channels such as the influenza matrix-2 (M2) protein (13), or proton ionophores (11). This led us to ask whether proton leakage from organelles is also involved in STING-induced LC3B lipidation and, if so, how STING activation leads to such ion transport.

## STING activation results in a pH increase in the Golgi

**Fig. 1.**
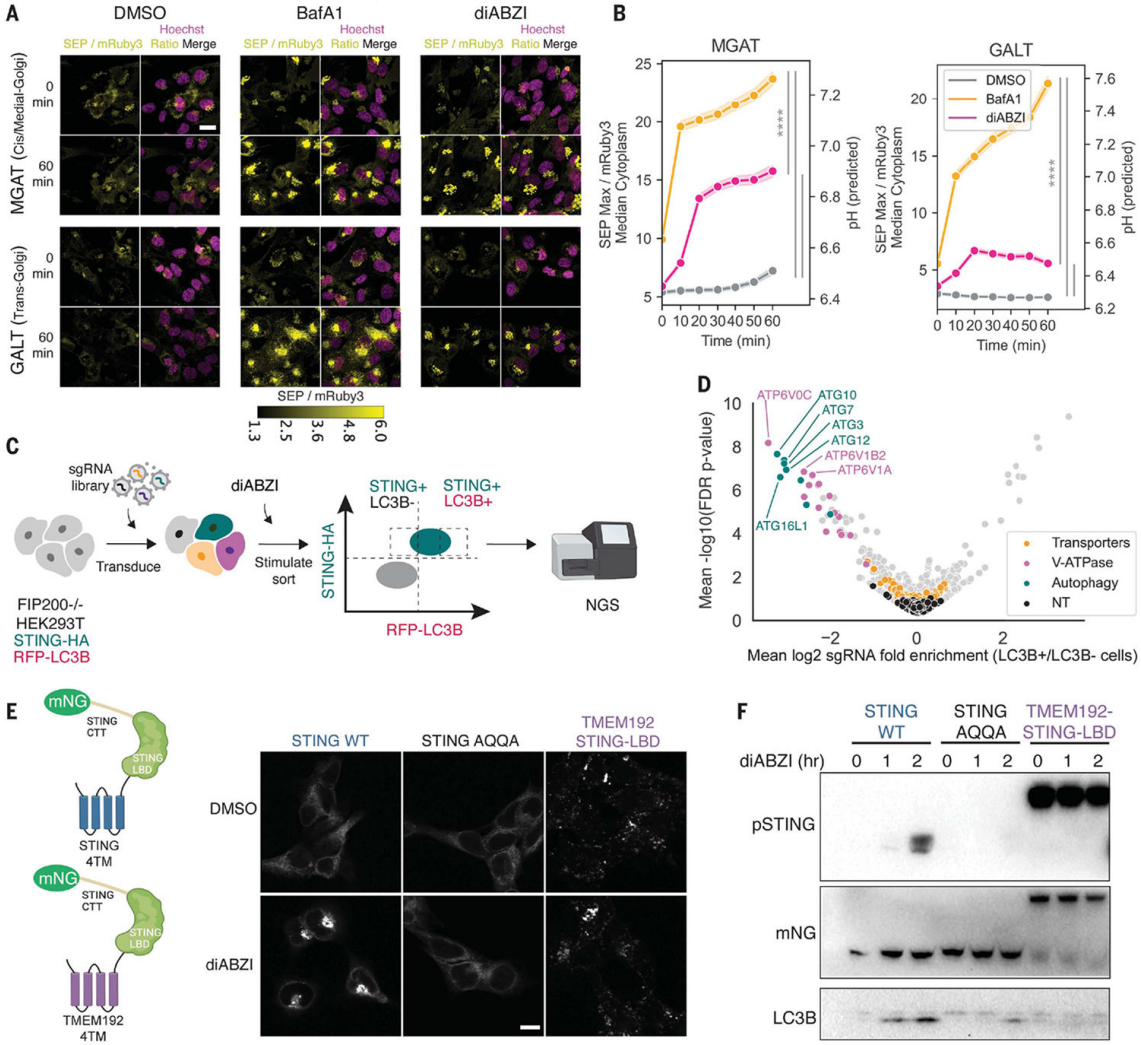
STING activation leads to a pH increase in the Golgi, and a genome-wide screen for regulators of STING-induced LC3B lipidation did not identify transporters that could mediate this effect. (**A**) Representative images of BJ1 cells expressing a ratiometric SEP and mRuby3 reporter localized to MGAT or GALT at 0 and 60 min after 1 μM diABZI or 1 μM BafA1 stimulation. Scale bar, 20 μm. (**B**) Quantification of experiment in (A); data were combined from three independent biological replicates. The pH was predicted with the linear regression model in fig. S1B. The shaded region denotes SD. One-way analysis of variance (ANOVA) followed by Tukey’s post hoc test at 60-min time point: ****P < 0.0001. (**C**) Workflow for the genome-wide CRISPR screen. (**D**) Volcano plot of genome-wide CRISPR screen results across two replicates; V-ATPase, noncanonical autophagy components, and known ion transporters (GO:0015075, ion transmembrane transporter activity) are highlighted. NT indicates nontargeting control single-guide RNAs. FDR, false discovery rate. (**E**) STING-mNeonGreen (mNG) constructs and representative images of STING mNG localization in 293T cells expressing WT STING, STING AQQA, or TMEM192-STING-LBD and stimulated with dimethyl sulfoxide (DMSO) or 1 μM diABZI for 1 hour. Scale bar, 10 μm. One representative experiment of *n* = 2 experiments. (**F**) Immunoblotting of phosphorylated STING (pSTING) and LC3B lipidation in 293T cells expressing WT STING, STING AQQA, or TMEM192-STING-LBD stimulated as in (E). One representative experiment of *n* = 3 experiments.

To test whether STING activation leads to proton transport out of acidic compartments, we constructed genetically encoded ratiometric pH sensors targeted to several organelles. As a sensor, we used superecliptic pHluorin (SEP), a variant of green fluorescent protein whose brightness increases with pH (14, 15), fused to pH-insensitive mRuby3. This ratiometric sensor was targeted to the cis/medial Golgi [through fusion to alpha-1,3-mannosyl-glycoprotein 2-beta-*N*-acetylglucosaminyltransferase (MGAT)], the trans Golgi [through fusion to galactose-1-phosphate uridylyltransferase (GALT)], or endolysosomes [through fusion to lysosomal-associated membrane protein 1 (LAMP1)] (16). These sensors were expressed in human BJ1 fibroblasts, and SEP-to-mRuby3 fluorescence ratios were correlated with intracellular pH values by using calibration data (fig. S1, A and B). Upon treatment with both the positive-control vacuolar adenosine triphosphatase (V-ATPase) inhibitor bafilomycin A1 (BafA1) and the STING agonist diamidobenzimidazole (diABZI), we observed that the ratio of SEP-to-mRuby3 fluorescence increased in both the cis/medial– and trans-Golgi compartments (Fig. 1, A and B, and fig. S1C). By contrast, in endolysosomal compartments, a pH increase was observed upon BafA1 treatment but not upon diABZI treatment (fig. S1, D and E). However, SEP has a p*K* ~7.1 (15), and our own pH calibration data showed low sensitivity to changes in pH <6.5 (fig. S1, A and B), so it remains possible that STING activation could elicit an endolysosomal pH increase that is below the sensor’s limit of detection.

## No known transporters mediate STING-induced LC3B lipidation

We next sought to systematically identify genes that mediate the pH increase observed in the Golgi compartment upon STING activation. Given that noncanonical LC3B lipidation is activated by proton leakage from acidic organelles, we reasoned that screening for genes that modulate STING-induced LC3B lipidation would also identify potential channel proteins responsible for the observed proton flux. We therefore carried out a genome-wide CRISPR fluorescence-activated cell sorting (FACS) screen, using human embryonic kidney 293T (HEK293T) cells transduced to express the autophagy-associated protein LC3B fused to red fluorescent protein (RFP) and hemagglutinin (HA)–tagged STING. To reduce the background lipidated LC3B signal derived from basal canonical autophagy, we knocked out FIP200 (10). After transduction with the Brunello genome-wide lentiviral library (17), cells were stimulated with the STING agonist diABZI and permeabilized to remove LC3B that was not lipidated, further reducing background fluorescence (18). STING-HA^+^ cells were sorted into LC3B^−^ and LC3B^+^ bins (Fig. 1C) to specifically identify STING-induced LC3B lipidation regulators that did not impair STING expression. The screen showed strong technical reproducibility (fig. S1, F and G, and table S1) and identified critical STING-induced LC3B lipidation regulators, including most of the known V-ATPase components, as well as noncanonical autophagy factors such as autophagy-related 16-like 1 (ATG16L1) (Fig. 1D). As a general mechanism of STING-induced LC3B lipidation, V-ATPase senses proton leakage from acidic vesicles through recruitment of V1 subunits to V0 complexes that together act as a scaffold for recruitment of ATG16L1, which initiates LC3B lipidation, through a process that is independent of V-ATPase’s proton-pumping function (9, 11, 12). Despite the high recovery rate of V-ATPase components and noncanonical autophagy factors, no other known channel protein perturbation significantly inhibited STING-dependent LC3B lipidation in our screen (Fig. 1D). We thus hypothesized that STING itself may mediate the observed Golgi proton leakage and thereby trigger V-ATPase assembly and subsequent recruitment of ATG16L1 to initiate LC3B lipidation (12).

To identify the domains of STING involved in LC3B lipidation, we first tested whether the STING ligand-binding domain (LBD), which has been proposed to recruit LC3B through its LC3-interacting region (LIR) motifs (5, 19), could induce LC3B lipidation upon translocation to Golgi or endosomes. We measured LC3B lipidation in 293T cells expressing wild-type (WT) STING, a STING oligomerization–deficient variant (A277Q/Q273A STING, termed “AQQA”) (20), or an endolysosome-localized STING fusion protein (the endolysosomal protein TMEM192 fused to the STING LBD) (21). After stimulation with the STING agonist diABZI, the AQQA variant exhibited impaired translocation, phosphorylation, and LC3B lipidation (Fig. 1, E and F). By contrast, TMEM192-STING-LBD did not induce LC3B lipidation despite its endolysosomal localization and strong induction of STING phosphorylation (Fig. 1, E and F). Because translocation of the STING LBD domain was not sufficient to induce LC3B lipidation, we hypothesized that STING’s transmembrane domain could play an important role in LC3B lipidation upon STING translocation.

## STING-mediated pH increase is inhibited by a small molecule binding a predicted pore in the transmembrane domain

Given the necessity of STING translocation from the ER to the Golgi for STING-induced LC3B lipidation and the known role of a pH increase in acidic organelles as a common trigger for this process, we considered whether STING could generate Golgi ion leakage by inducing membrane damage, resulting in a secondary ion leakage, or by directly acting as an ion channel through its transmembrane domain. STING translocation is known to induce LC3B lipidation without formation of galectin-3 puncta (9), suggesting that STING activation does not result in membrane damage. We thus investigated whether STING directly acts as a channel for proton release into the cytosol upon translocation to the Golgi, an acidic compartment (16). STING-dependent induction of LC3 lipidation is an ancestral function of the sensor conserved from *Homo sapiens* to *Nematostella vectensis* (5). Thus, if STING functions as a channel, this activity should be structurally conserved.

**Fig. 2.**
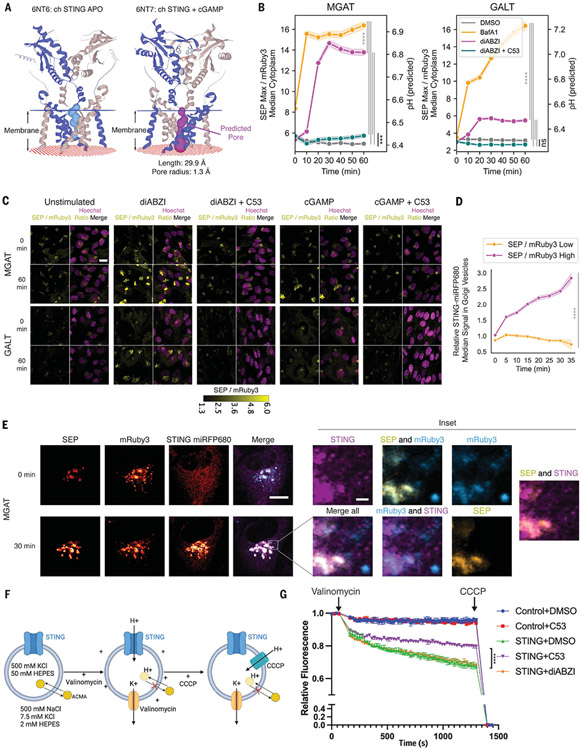
A pore-binding small molecule inhibits pH increase in cells stimulated with STING agonist, and STING transports protons in an in vitro liposome assay. (**A**) Predicted pore for chicken cGAMP–bound STING but not the apo conformation; key parameters were calculated with MOLEonline. (**B**) Quantification of pH increase in BJ1 cells from 0 to 60 min after 1 μM diABZI or 1 μM BafA1 stimulation with or without 10 μM C53; data from three biological replicates were combined. The shaded region denotes SD. One-way ANOVA followed by Tukey’s post hoc at the end point measurements: ***P < 0.001; ****P < 0.0001; n.s., not significant, *P* > 0.05. (**C**) Representative images of BJ1 cells in (B) and fig. S2b. Scale bar, 20 μm. (**D**) Quantification of super-resolution Airyscan images of BJ1 MGAT SEP mRuby3 STING knockout cells overexpressing STING WT miRFP680 stimulated with 1 μM diABZI, representing four biological replicates and five individual cells. STING intensity was normalized to the per-cell baseline median intensity. Shaded region denotes SD. Two-tailed Student’s *t* test at the end point measurements: ****P < 0.0001. (**E**) Representative super-resolution Airsycan images of BJ1 cell from (D) at 0 min and 30 min after 1 μM diABZI stimulation. Scale bar, 10 μm; inset scale bar, 1 μm. (**F**) Schematic of the ACMA-based fluorescence flux assay. (**G**) ACMA-based fluorescence influx assay performed using preformed liposomes loaded with STING protein (protein:lipid at a 1:200 mass ratio) or matched detergent micelle containing buffer (Control). Loaded liposomes were treated with DMSO, 100 μM C53, or 1 μM diABZI. One representative experiment of *n* = 4 experiments carried out with two distinct batches of purified STING protein. Error bars indicate SD. Two-way ANOVA followed by Tukey’s post hoc at the end point measurements: ****P < 0.0001; n.s., *P* > 0.05. For multiple comparisons, only “STING + diABZI” versus “STING + DMSO” and “Control + DMSO” versus “Control + C53” have n.s. *P* value; comparisons between other groups all have *P* < 0.0001.

To investigate whether STING could function as an ion channel, we analyzed published cryo–electron microscopy (cryo-EM) structures of chicken STING (20) with MOLEonline, a tool for automated detection and characterization of channels in macromolecules (22). When we analyzed the cryo-EM structures of ligand-free STING [Protein Data Bank (PDB) structure: 6NT6] compared with STING bound to its native ligand, cGAMP (PDB structure: 6NT7), the tool suggested a pore spanning the lipid bilayer (1.3-Å bottleneck radius, 29.9-Å length) in ligand-bound STING that was absent in ligand-free apo STING; the latter showed a central cavity that did not span the whole membrane (Fig. 2A and fig. S2A). A recently discovered STING agonist, compound 53 (C53) (23), binds to the STING transmembrane domain in the area of the putative pore. We hypothesized that C53 could be used as a tool for inhibition of the proposed ion-channel function of STING. Indeed, the STING-mediated Golgi pH increase observed upon treatment with agonists diABZI or cGAMP alone was significantly reduced when cells were cotreated with C53 along with diABZI or cGAMP (Fig. 2, B and C; fig. S2B; and movie S1), which is consistent with the idea that the proposed pore region of STING is required to produce a pH change within the Golgi. To exclude a potential role for downstream noncanonical autophagy factors in mediating the observed pH increase, we knocked out ATG16L1, which is essential for STING-induced LC3B lipidation (10), in BJ1 pH reporter cells. As expected, we found no significant inhibition of STING agonist–induced Golgi-pH increase in these cells (fig. S2, C and D). To further confirm STING’s mediation of the observed pH increase, we knocked out endogenous STING in BJ1 cells expressing the cis/medial–Golgi pH reporter (fig. S2E) and overexpressed STING-miRFP680. We then stimulated these cells with diABZI and used live-cell super-resolution Airyscan imaging to image STING translocation to individual Golgi vesicles. STING preferentially translocated to cis/medial–Golgi vesicles with a pH increase, indicated by higher SEP relative to the mRuby3 signal (Fig. 2, D and E, and movie S2). Quantification revealed an increase in STING signal over time in vesicles with a high SEP-to-mRuby3 ratio, with little change in STING intensity at vesicles with a low SEP-to-mRuby3 ratio (Fig. 2D), as well as an increase in the correlation between STING- and vesicle-SEP-to-mRuby3 ratio over time (fig. S2F), as expected if STING translocation to acidic Golgi compartments induces proton leakage.

## STING reconstituted on liposomes transports protons

To explore the sufficiency of STING for mediating proton transport, we purified full-length human STING (fig. S2G) (20) and reconstituted STING into liposomes (24-26) (fig. S2H). We used the pH-sensitive dye 9-amino-6-chloro-2-methoxyacridine (ACMA) to measure proton flux into liposomes. ACMA will be sequestered in liposomes and its fluorescence quenched upon pH changes induced by proton transport from the external buffer into the vesicles, enabling quantification of proton flux on the basis of a reduction in total ACMA fluorescence (Fig. 2F). Proton flux was observed in STING proteoliposomes and was reduced in the presence of C53, whereas control liposomes formed with identical solutions devoid of protein did not show proton flux (Fig. 2G). Thus, STING appears to be sufficient to transport protons across lipid membranes. To further control for potential effects of detergent, we removed detergent with Bio-Beads (Bio-Rad), which did not reduce proton flux by STING proteoliposomes but did reduce proton leakage induced by addition of high (30 times as much as the standard amount) (fig. S2I) detergent concentrations (fig. S2J). In contrast to the behavior in live cells, where STING-mediated proton leakage was induced by STING agonists such as diABZI or cGAMP, in liposomes, STING mediated proton leakage similarly in the presence or absence of diABZI (Fig. 2G). The dispensability of diABZI for proton flux in this reductionist liposome assay suggests that a voltage difference or pH gradient (such as found in the Golgi) could induce an open conformation of STING and enable proton transport. Agonist binding in cells would thus appear mainly to be required for translocation of STING to this acidic organelle. By contrast, C53 directly reduces STING-driven proton transport in vitro.

## STING’s channel activity is required for its induction of LC3B lipidation

**Fig. 3.**
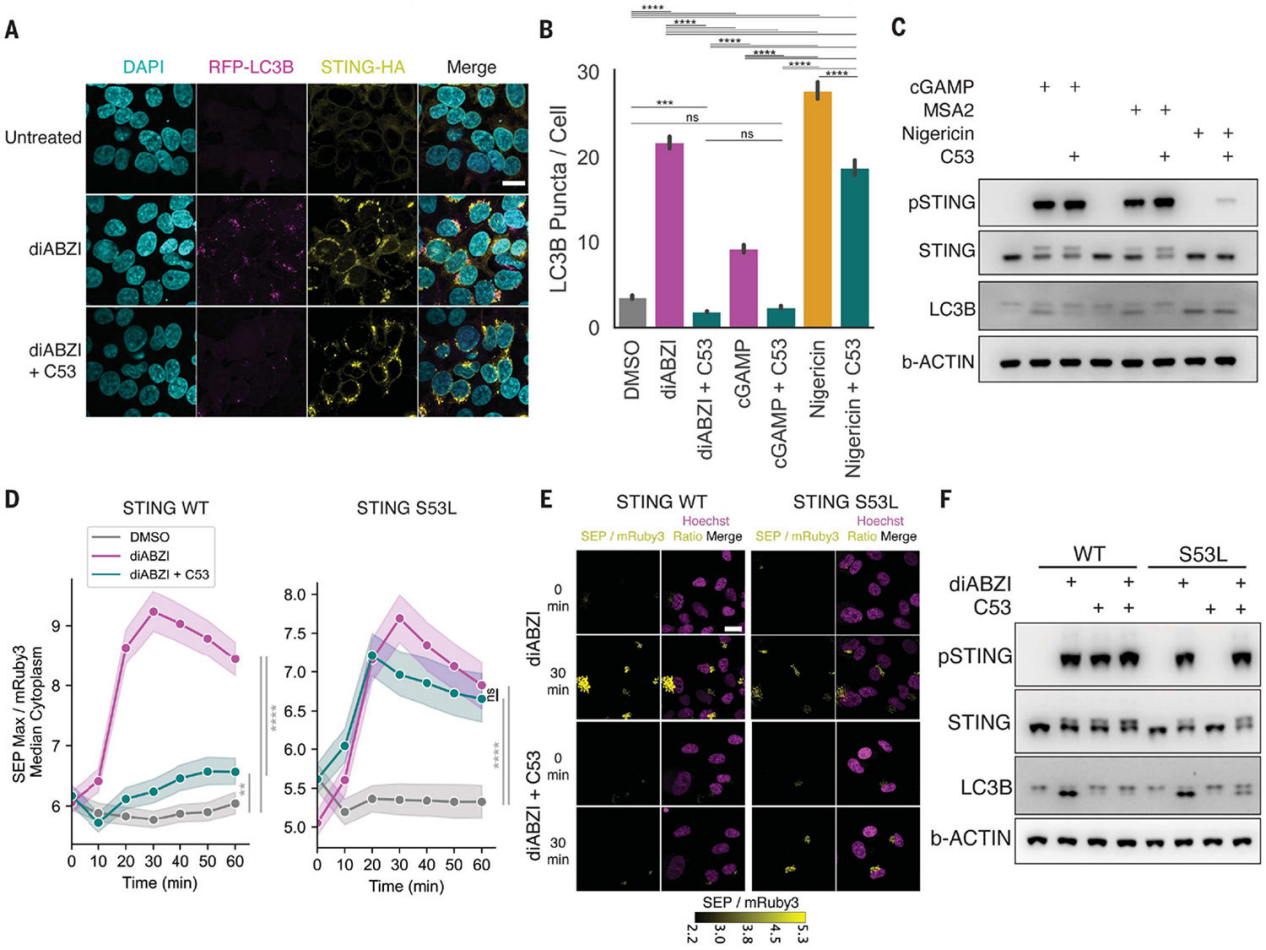
STING-induced LC3B lipidation is inhibited by C53, and STING S53L is less sensitive to C53-mediated inhibition of pH increase and LC3B lipidation. (**A**) Representative images of stably expressed RFP-LC3B and STING-HA in FIP200 KO 293T cells upon 1 μM diABZI stimulation for 1 hour with or without 10 μM C53 cotreatment. Scale bar, 20 μm. DAPI, 4′,6-diamidino-2-phenylindole. (**B**) Quantification of experiment in (A), representing three biological replicates combined. Error bars indicate SD. One-way ANOVA followed by Tukey’s HSD: ***P < 0.001; ****P < 0.0001; n.s., *P* > 0.05. (**C**) Immunoblots for indicated proteins in BJ1 cells with or without cotreatment with 10 μM C53 upon 20 μg/ml cGAMP (permeabilized with 5 μg/ml digitonin), 40 μM MSA-2, or 2 μM nigericin stimulation. One representative experiment of n = 3 experiments. (**D**) Quantification of pH change from 0 to 60 min after 1 μM diABZI stimulation with or without 10 μM C53; data from three biological replicates were combined. STING was knocked out in BJ1 cells followed by overexpression of STING WT (left) or STING S53L (right). Shaded region denotes SD. One-way ANOVA followed by Tukey’s post hoc at the end point measurements: **P < 0.01; ****P < 0.0001; n.s., *P* > 0.05. (**E**) Representative images of BJ1 cells assayed in (D). Scale bar, 20 μm. (**F**). Immunoblots of indicated proteins in 293T cells expressing STING WT or STING S53L treated with 1 μM diABZI with or without 10 μM C53 for 1 hour. One representative experiment of *n* = 3 experiments.

Given the observed impairment of STING-mediated ion leakage upon treatment with C53 both in cells and in vitro, we next asked whether C53 could inhibit other downstream functions of STING activation. We first tested whether STING-induced LC3B lipidation could also be inhibited by C53. Indeed, treatment with both cGAMP and noncyclic dinucleotide agonists MSA-2 or diABZI induced LC3B lipidation, whereas cotreatment with C53 strongly impaired LC3B lipidation without associated inhibition of STING phosphorylation or STING translocation (Fig. 3, A to C, and fig. S3, A and B). C53 cotreatment did not greatly inhibit LC3B lipidation induced by nigericin, an ionophore that induces noncanonical LC3B lipidation independently of STING (27) (Fig. 3, B and C), suggesting that C53’s activity is specific to STING-dependent LC3B lipidation. To further exclude a STING-independent effect for C53, we knocked out endogenous STING in BJ1 cells expressing the cis/medial–Golgi pH reporter and overexpressed WT STING or STING S53L (fig. S3C), a STING variant with reduced binding to C53 (23). We then measured pH changes upon stimulation with diABZI and observed that C53 cotreatment inhibited agonist-mediated pH increases in cells expressing WT STING but had no significant effect in cells expressing STING S53L (Fig. 3, D and E). Similarly, 293T cells stably transduced with STING S53L exhibited reduced sensitivity to C53-mediated impairment of LC3B lipidation induced by diABZI treatment relative to cells expressing WT STING (Fig. 3F).

## STING’s channel activity is required for its activation of the NLRP3 inflammasome

**Fig. 4.**
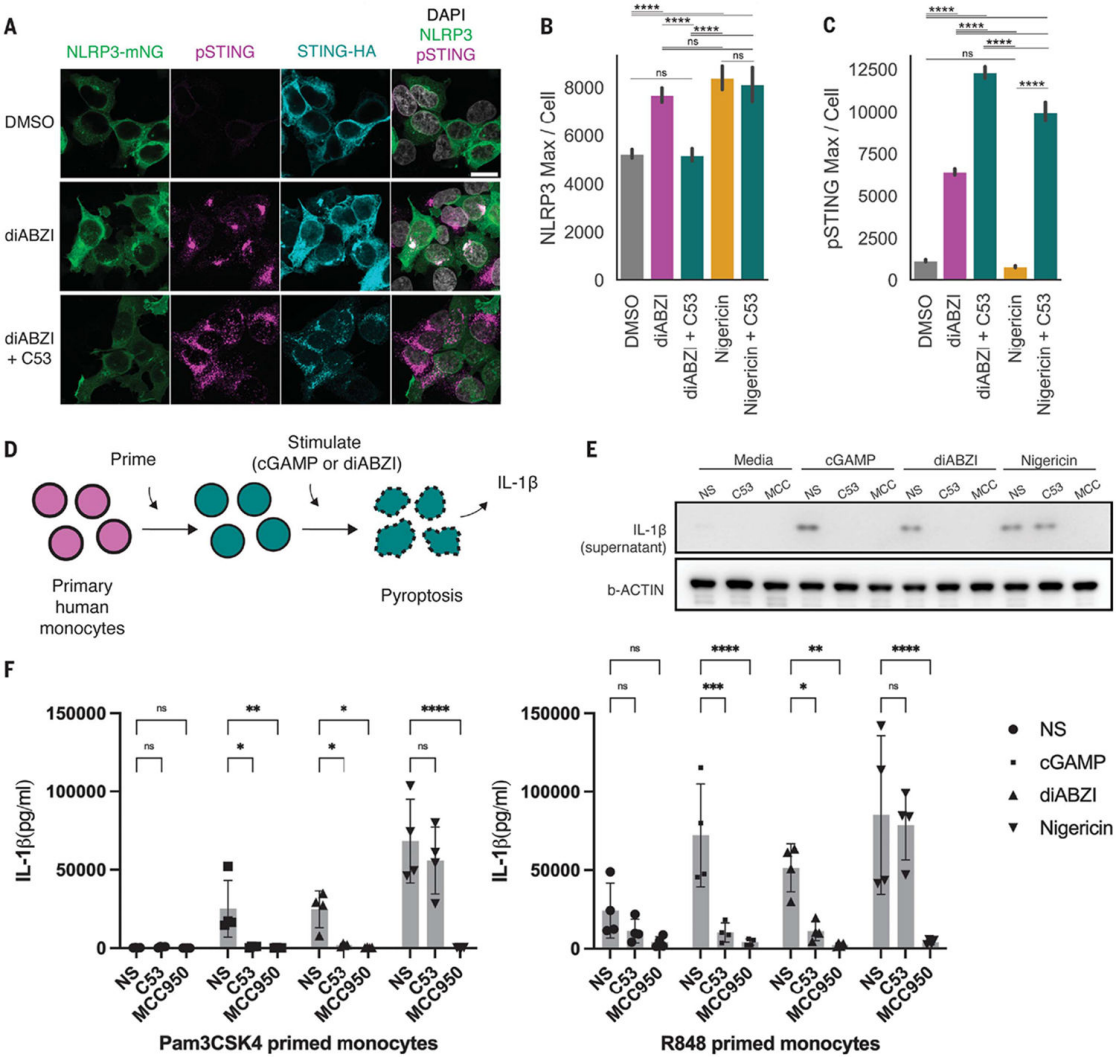
STING-induced NLRP3 inflammasome activation and IL-1β release are inhibited by C53. (**A**) Representative images of pSTING, STING, and NLRP3 in HEK293T cells expressing STING-HA and NLRP3-mNeongreen (NLRP3-mNg) treated with DMSO and 1 μM diABZI with or without 10 μM C53 for 1 hour. Scale bar, 20 μm. (**B**) NLRP3 translocation quantified as the per-cell maximum NLRP3 intensity from experiment in (A) from three biological replicates combined. One-way ANOVA followed by Tukey’s post hoc: ****P < 0.0001; n.s., *P* > 0.05. (**C**) Same as (B) but quantifying pSTING intensity. (**D**) Experimental workflow for inflammasome induction in primary human monocytes. (**E**) Immunoblots of processed IL-1β from human monocytes (primed with R848) upon no stimulus (NS), 10 μg/ml cGAMP, 1 μM diABZI, or 6.7 μM nigericin stimulation in the absence or presence of 10 μM C53 or 5 μM NLRP3 inhibitor MCC950 (MCC). One representative donor of *n* = 3 donors tested. (**F**) Supernatant cytokine measurement from stimulated human monocytes [(left) Pam3CSK4 primed, (right) R848 primed] of processed IL-1β upon NS, 10 μg/ml cGAMP, 1 μM diABZI, or 6.7 μM nigericin stimulation in the absence or presence of 10 μM C53 or the 5 μM NLRP3 inhibitor MCC950; each data point represents one donor with total *n* = 4 donors. Error bars indicate SD. Two-way ANOVA followed by Dunnett’s multiple comparisons test: n.s., *P* > 0.05; *P < 0.05; ***P < 0.001; ****P < 0.0001.

In addition to induction of interferon and LC3B lipidation, STING activates the NLRP3 inflammasome in human myeloid cells (6), but the mechanism remains unclear. The influenza virus M2 pore protein induces noncanonical LC3B lipidation by inducing proton leakage (13), while also activating the NLRP3 inflammasome (28). Perhaps then STING might activate the inflammasome in a similar manner, and C53 could block this activity. Upon activation, NLRP3 translocates from the cytosol to Golgi vesicles, where it initiates downstream inflammasome activation (29). Using an NLRP3-mNeonGreen reporter, we found that NLRP3 formed puncta upon stimulation with the STING agonist diABZI (Fig. 4A), similarly to when cells were stimulated with the NLRP3 agonist nigericin (fig. S4A) (29). Furthermore, NLRP3 colocalized with STING and phosphorylated STING (pSTING) on these puncta (Fig. 4A). Consistent with the hypothesis that STING-induced proton leakage is the driver of downstream NLRP3 activation, when we treated cells with both diABZI and C53, we observed a significant reduction in NLRP3 translocation together with an enhancement in STING phosphorylation (Fig. 4, B and C). We also tested whether STING-induced LC3B lipidation could have a role in STING-induced inflammasome activation by knocking out ATG16L1 in BLaER1 cells (fig. S4B), a human cell line that can be transdifferentiated to monocytes and in which STING activation leads to NLRP3-dependent interleukin-1β (IL-1β) release (6). Knockout of ATG16L1 did not impair diABZI-induced IL-1β release (fig. S4C). Thus, STING-induced inflammasome activation is independent from STING-induced LC3B lipidation. Lastly, we tested whether C53 could block STING-induced inflammasome activation as measured by IL-1β release and cell death in primary CD14^+^ monocytes (Fig. 4D). In agreement with our findings in HEK293T cells, C53 cotreatment significantly impaired STING-induced inflammasome activation, inhibiting IL-1β release (Fig. 4, E and F) and cell death (fig. S4D) in primary human monocytes stimulated with cGAMP or diABZI to a level similar to that of monocytes treated with the NLRP3 inhibitor MCC950. C53 did not affect IL-1β release (Fig. 4, E and F) or cell death (fig. S4, C and D) when the NLRP3 inflammasome was activated by nigericin, further indicating that C53 impairs activation of the NLRP3 inflammasome in a STING-specific manner. Thus, similarly to the influenza protein M2, STING activates the NLRP3 inflammasome through induction of a proton leakage.

## Discussion

Here we demonstrated that STING activation induces proton leakage at the Golgi through a channel formed at the interface of the STING homodimer’s transmembrane domains. This STING-mediated pH increase is inhibited by the small molecule C53, whereas no inhibition of pH increase was observed in cells expressing STING S53L, a STING variant with reduced binding to C53 (23). We also showed that STING transports protons in an in vitro liposome assay and that C53 treatment inhibited STING proton transport in vitro.

In addition to demonstrating proton transport through purified STING in vitro and STING-dependent pH changes in cells, we also found that STING’s channel activity is critical for downstream activation of LC3B lipidation and of the NLRP3 inflammasome because treatment with C53 impaired these activities without reducing STING phosphorylation. C53 treatment also inhibited NLRP3 inflammasome activation downstream of STING in primary human monocytes. These findings provide an avenue for decoupling STING phosphorylation from induction of LC3B lipidation and inflammasome activation induced by STING. Comparing the effects of agonists that bind STING’s natural binding pocket or a pore-associated pocket, such as C53, could help determine the relative importance of STING phosphorylation versus channel-mediated downstream functions in diverse biological contexts and therapeutic applications.

## Data and materials availability:

All screen results are contained in the supplementary materials. Image analysis code is available on GitHub at https://github.com/liucarlson2023/STINGChannel and Zenodo (30). Example images [three uncropped fields of view for each of three replicates for all imaging experiments (seven total datasets), as well as all crops used in the manuscript] are available at Zenodo (datasets summarized in table S3). Reporter plasmids have been deposited to Addgene (plasmid numbers summarized in table S4).

## Supplementary Material

S2 Video

S1 Video

Table S1

Supplementary Material

Reproducibility_checklist
