# Supplementary material for: Human STING is a proton channel
Source: Science. Author manuscript; Available in PMC 2024 Jul 21. (PMC11260435; doi:10.1126/science.adf8974)
Supplement: Supplementary Material [file NIHMS2002534-supplement-Supplementary_Material.pdf]

Science
AAAS

Supplementary Materials for

Human STING is a proton channel

Bingxu Liu *et al*.

Corresponding authors: Bingxu Liu, bggscientific@gmail.com; Darrell J. Irvine, djirvine@mit.edu; Nir Hacohen, nhacohen@mgh.harvard.edu

Science **381**, 508 (2023)
DOI: 10.1126/science.adf8974

The PDF file includes:

Materials and Methods
Figs. S1 to S4
Tables S2 to S4
References

Other Supplementary Material for this manuscript includes the following:

Table S1
Movies S1 and S2
MDAR Reproducibility Checklist

Materials and Methods

Cell lines and constructs

293T (CRL-3216) and hTert-BJ1 (BJ-5ta - CRL-4001) were from ATCC. 293T and BJ1 were cultured in DMEM (Corning) supplemented with 10% FBS (VWR), 1X GlutaMax (Thermo Fisher) and 1X Penicillin/Streptomycin (Corning). The plasmids psPAX2 (#12260) and pCMV-VSV-G (#8454) were from Addgene. pTRIP-SFFV/PGK-Blast-2A-STING-HA was from an earlier publication(*21*). pTRIP-SFFV-Blast-2A-STING (S53L)-HA were cloned through PCR primers containing mutant sequences. pTRIP-SFFV-Blast-2A-NLRP3-mNG and pTRIP-SFFV-Hygro-2A-RFP-LC3B were cloned from Gibson assembly using gBlocks. Plasmids for pH imaging are described in detail in the "Organelle pH Measurements and Image Acquisition" section. *FIP200* KO 293T cells used for genetic screens were generated by transfecting 293T cells with pXPR_023 (Addgene #52961), encoding Cas9 and an sgRNA against *FIP200* (*21*) and selecting cells with 2 µg/mL puromycin (Thermo Fisher Scientific #A1113803) for 2 days. *FIP200* KO 293T cells were then transduced with pTRIP-PGK-Hygro-P2A-RFP-LC3B, treated with 33 µg/mL hygromycin (Invivogen, #ant-hg-1) for 3 days and then sorted to obtain a homogenous RFP+ population.

Genome-wide CRISPR screen

RFP-LC3B *FIP200* KO 293T cells were transduced with pTRIP-PGK-Blast-P2A-STING-HA and selected with 20 µg/mL blasticidin (Thermo Fisher Scientific #A1113903). For screening, 200M RFP-LC3+ STING-HA+ *FIP200* KO 293T cells were transduced with Cas9-sgRNA all-in-one Brunello library at MOI=0.4 and selected with 2 µg/mL puromycin for 2 days. 8 days after transduction, 200M transduced cells were treated with 1 µM diABZI (Invivogen, #tlrl-diabzi) for 2.5hr, and then permeabilized with 1X perm buffer (PBS with 0.05% saponin and 0.1% glycine) for 4 min (*18*). Permeabilized cells were then washed with 1X PBS, and fixed, permeabilized further, and stained using BD Cytofix/Cytoperm kit (#554714) following the instruction with anti-HA Alexa647 (Biolegend, #682404) 1:200 dilution.

Human STING purification

The process described here is adopted from a previous study(*20*). Briefly, Expi293F cells were transduced with SFFV-Blast-P2A-STING-FLAG and culture in Expi293 media (Thermo) with density between 4-6 million/mL for harvest. For each batch of protein purification, 3 L of cells were pelleted and resuspended in 50 mL buffer A (20 mM Tris-HCl pH 8.0, 150 mM NaCl, with protease inhibitor (Roche)), and then sonicated (20s total, 50% on time, 5 power using a Branson digital sonicator, repeated 3 times to break down all cells efficiently). Sonicated cells were centrifuged at 5000 g for 10 minutes to remove debris, and all the supernatant was collected and ultracentrifuged (100,000 g, 1 hour using Beckman Optima XPN90). After removing the supernatant, the pellets were resuspended with 25 mL Buffer A with 1.5% 10:1 DDM/CHS (Anatrace, D310-CH210), and then centrifuged at 5000 g, 10 min to remove all insoluble debris. The supernatant was collected and added to 1 mL of Buffer A pre-washed FLAG M2 beads (Sigma, 2426-1ML or M8823) and incubated at 4ºC for 1 hr. Beads were then washed with wash buffer (25 mM Tris-HCl pH 8.0, 300 mM NaCl, 10:1 DDM/CHS 0.1%) 3 times and then eluted with 0.5 mL elution buffer (25 mM Tris-HCl pH 8.0, 200 mM NaCl, 10:1 DDM/CHS 0.1%, 0.6 mg FLAG peptide (Sigma)) at 4ºC for 3 hr. Eluted samples were loaded on an FPLC (AKTA) to perform Size Exclusion Chromatography (SEC) using a Superdex 200 increase 10/300 column (Cytiva) using a running buffer (25 mM Tris-HCl pH 8.0, 150 mM NaCl, 10:1 DDM/CHS 0.03%).

Liposome synthesis.

Liposomes were generated via the thin film hydration method. Briefly, a mixture of POPE (1-palmitoyl-2-oleoyl-sn-glycero-3-phosphoethanolamine) and POPG [1-palmitoyl-2-oleoyl-sn-glycero-3-phospho-(1'-rac-glycerol)] (3:1, wt/wt) in chloroform (Avanti Polar Lipids) was dried in a rotovap (Rotovapor RII, BUCHI) to generate a lipid film (*24*). The film was hydrated with a high potassium buffer (500 mM KCl, 50 mM HEPES, pH 7.3) at 100 mg/mL and sonicated at 65°C until monodisperse liposomes were formed. Liposomes underwent 7 cycles of freeze-thawing and were sonicated in a water bath until they reached 200-300 nm in size. Size was measured via dynamic light scattering using a Zetasizer Nano ZSP (Malvern).

Protein incorporation into lipid vesicles.

STING was loaded onto preformed liposomes as described previously for other channel proteins (*31*, *32*). Briefly, liposomes were diluted to 10 mg/mL using a low potassium buffer (500 mM NaCl and 2 mM HEPES, pH 7.3), then mixed with purified STING protein on detergent micelles at a detergent concentration of ~0.02%. The detergent concentration was above its critical micelle concentration (CMC) to enable protein loading onto liposomes, but not sufficient to disrupt the lipid bilayer (*26*, *32*). Samples were then allowed to incubate for 30 minutes prior to assaying for proton flux. Control liposomes were generated via the same protocol, but were mixed only with the same buffer containing empty detergent micelles.

Proteoliposome proton flux assay.

The proton flux assay was performed following previously established protocols (*32*). Liposomes or proteoliposomes were diluted to 1 mg/mL or less in the low potassium buffer with 2 µM of ACMA (9-Amino-6-Chloro-2-Methoxyacridine, Thermo Fisher #A1324). The ACMA fluorescence (excitation at 419 nm and emission at 490 nm) was measured on a black 384-well plate for at least 1 minute to obtain a baseline fluorescence on an Infinite M200 pro (TECAN) plate reader. To initiate proton flux, 0.04 µM of valinomycin (Thermo Fisher, #V1644) was added to each sample and mixed. Fluorescence readings were then performed at 20-30 second intervals until no substantive changes were further observed (~10-15 minutes). To terminate the assay, 4 µM of CCCP (carbonyl cyanide 3-chlorophenylhydrazone, Sigma #C2759) was added to each sample and mixed and the fluorescence readings were measured for at least 1 minute. Relative fluorescence was calculated based on the difference between the measured fluorescence and the final fluorescence relative to the difference between the initial fluorescence and the final fluorescence. The same process was performed on the control liposomes.

Detergent removal using Bio-Beads

In addition to performing the experiment with control liposomes, we used Bio-Beads (Bio-Rad Bio-Beads SM-2) to extract any residual detergent in our proteoliposome preparations. Beads were prepared as described previously (*33*). Three rounds of fresh beads were added to proteoliposomes at a concentration of 30 mg/mL for 30 minutes under shaking.

Organelle pH Measurements and Image Acquisition

hTERT-immortalized BJ1 cells (ATCC CRL-2522) were transduced with lentiviral ratiometric reporters targeted to MGAT, GALT, or LAMP1 constructed based on previously reported designs (*16*) (modified from Addgene plasmid # 171718, Addgene plasmid # 171719, and Addgene plasmid # 171720) with superecliptic pHluorin (Addgene plasmid # 32001) and mRuby3 (Addgene plasmid #127808). Transduced cells were sorted based on mRuby3 expression using a Sony MA900 sorter. For imaging assays, BJ1 SEP mRuby3 cells were plated in 24-well glass-

bottom plates (Greiner Bio-One) at 40,000 cells/well. After 48 hours, cells were stained for 45 minutes at 37°C with 0.5 µg/ml Hoechst 34580 (Thermo Fisher Scientific, cat. #H21486). Cells were then washed and incubated in Fluorobrite DMEM (Thermo Fisher Scientific, cat. #A1896701) medium supplemented with 10% FBS, 1% Pen-strep, and 1x GlutaMAX (Thermo Fisher Scientific, cat. #35050061). For pH calibration experiments, cells were treated with buffers at defined pH values supplemented with nigericin and valinomycin following manufacturer's instructions (Thermo Fisher Scientific cat. # P35379). Additional buffers at pH 7, 6, and 5 were created by titration with HCl. For time-course experiments, cells were stimulated with 1 µM Bafilomycin A1 (Santa Cruz Biotechnology cat. #sc-201550), 1 µM diABZI (Invivogen, #tlrl-diabzi), or 20 µg/mL cGAMP (Invivogen, #tlrl-nacga23-1) with 5 ng/µL digitonin (Promega, #G9441) for 1 hr with or without the addition of 10 µM C53 (Cayman, #37354). For experiments assaying the effect of STING S53L, hTERT-immortalized BJ1 cells expressing SEP mRuby3 targeted to cis/medial Golgi (MGAT) were transduced with pXPR023 (lentiCRISPRv2) expressing an sgRNA targeting STING and selected with 0.1 µg/mL puromycin for 5 days. Cells were then transduced with blasticidin-STING-HA (WT or S53L) and selected using 10 µg/mL blasticidin HCl for 5 days. All images were acquired using a Ti2-E inverted epifluorescence microscope (Nikon) with automated XYZ stage control, hardware autofocus, and a Yokogawa CSU-W1 confocal spinning disk unit with Zyla 4.2 PLUS sCMOS camera. An Okolab cage incubator was set to 37°C with 5% CO_2. 405, 488, 561, and 640 nm laser lines were used for fluorescence illumination and all hardware was controlled using NIS Elements software. Images were acquired using a 40X 0.95 NA CFI Plan Apo λ objective (Nikon MRD70470) with the following lasers and filters: Hoechst (405 nm laser, Chroma Multi LED set #89401), superecliptic pHluorin (488 nm laser, Chroma Multi LED set #89401), and mRuby3 (561 nm laser, Chroma Multi LED set #89401), assaying three z planes per field of view with 1.25 µm spacing. Fields of view were selected using NIS Elements software coordinates without manual preselection.

Autophagy Induction and Image Acquisition

RFP-LC3B and STING-HA-expressing FIP200 KO 293T cells were seeded on Fibronectin bovine plasma coated 24-well glass-bottom plates (Greiner Bio-One) the night before stimulation. Cells were then stimulated with 20 µg/ml cGAMP (Invivogen, #tlrl-nacga23-1) with 5 µg/ml digitonin (Promega, #G9441) or 1 µM DIABZI (Invivogen, #tlrl-diabzi) with or without the addition of 10 µM C53 (Cayman, #37354) for 1 hour. Cells were then fixed with 2% Paraformaldehyde (Electron Microscopy Sciences, #15710) in PHEM buffer (Electron Microscopy Sciences, #11162) for 30 minutes at 37°C, washed three times with PBS and quenched with freshly prepared 0.1M Glycine (Sigma, #50046) for 10 minutes. Cells were permeabilized in 100% methanol for 30 minutes and stained with anti-HA (Millipore, #11867423001) for 1 hour at room temperature in 3% BSA, washed 5 times, and then stained with Alexa 647 anti-rat IgG (H+L) (Thermo, A-21247) in 3% BSA for 1 hour. After five washes, cells were incubated in 2X SSC with 200 ng/mL DAPI (Thermo Fisher) and imaged using the Nikon microscope used for organelle pH images. Images were acquired using a 60X 1.40 NA Plan Apo λ oil immersion objective (Nikon MRD01605) with Nikon type F immersion oil with the following lasers and filters: DAPI (405 nm laser, Chroma ET455/50), RFP-LC3B (561 nm laser, Chroma ET605/52), and STING-HA (640 nm laser, Chroma ET705/72), assaying five z planes per field of view with 0.625 µm spacing. Fields of view were selected using NIS Elements software coordinates without manual preselection.

NLRP3 Stimulation and Image Acquisition

HEK293T cells transduced to express NLRP3-mNeonGreen and STING-HA were plated in 24-well glass-bottom plates (Greiner Bio-One) and, after 24 hours, stimulated with 2 µM nigericin or

1 µM diABZI with or without the addition of 10 µM C53 for 1 hour. Cells were then fixed with 2% Paraformaldehyde (Electron Microscopy Sciences, #15710) in PHEM buffer (Electron Microscopy Sciences, #11162) for 30 minutes at 37°C, washed three times with PBS and quenched with freshly prepared 0.1M Glycine (Sigma, #50046) for 10 minutes. Cells were permeabilized in 100% methanol for 30 minutes and stained with anti-HA (Millipore, #11867423001) and anti p-STING (Cell Signaling Technology cat. #19781s) for 1 hour at room temperature in 3% BSA, washed 5 times, and then stained with Alexa 647 anti-rat IgG (H+L) (Thermo, A-21247) and Alexa 555 plus anti-rabbit (Thermo, A32732) in 3% BSA for 1 hour. After five washes, cells were incubated in 2X SSC with 200 ng/mL DAPI (Thermo Fisher) and imaged using the Nikon microscope used for organelle pH images. Images were acquired using a 60X 1.40 NA Plan Apo λ oil immersion objective (Nikon MRD01605) with Nikon type F immersion oil with the following lasers and filters: DAPI (405 nm laser, Chroma ET455/50), NLRP3 mNeonGreen (488nm laser, Chroma ET525/36) pSTING (561 nm laser, Chroma ET605/52), and STING-HA (640 nm laser, Chroma ET705/72), assaying five z planes per field of view with 0.625 µm spacing. Fields of view were selected using NIS Elements software coordinates without manual preselection.

Super-resolution Airyscan Imaging

hTERT-immortalized BJ1 cells (ATCC CRL-2522) expressing SEP mRuby3 targeted to cis/medial Golgi (MGAT) were transduced with pXPR023 (lentiCRISPRv2) expressing an sgRNA targeting STING and selected with 0.1 µg/mL puromycin for 5 days. Cells were then transduced with blasticidin-STING-miRFP680 (constructed using Addgene plasmid # 136557) and selected using 10 µg/mL blasticidin HCl for 5 days. Cells were plated in 96-well glass-bottom plates (Greiner Bio-One) at 6,000 cells/well. After 48 hours, cells were incubated in Fluorobrite DMEM (Thermo Fisher Scientific, cat. #A1896701) medium supplemented with 10% FBS, 1% Pen-strep, and 1x GlutaMAX (Thermo Fisher Scientific, cat. #35050061) and stimulated with 1 µM diABZI (Invivogen, #tlrl-diabzi). All images were acquired using an LSM980 with Airyscan2 (Zeiss) with 37°C and 5% CO_2 incubation. 8 z-stacks were acquired with 0.15 µm z-step. Images were acquired using a 63X 1.40 NA DIC M27 objective with Immersol 518F 37°C oil. Acquired images were Airyscan processed and then analyzed as described in the image analysis section.

Immunoblotting:

Cells were seeded at 0.15 million cells (BJ1) or 0.2 million cells (293T) per well in a 24 well plate the night before stimulation. Cells were then stimulated with 1 µM diABZI (Invivogen, #tlrl-diabzi), 2 µM Nigericin (Invivogen, #tlrl-nig), 20 µg/mL cGAMP (Invivogen, #tlrl-nacga23-1) with 5 µg/mL digitonin (Promega, #G9441) or 40 µM MSA2 (Invivogen, #tlrl-diabzi) with or without the addition of 10 µM C53 (Cayman, #37354) for 1 hour. Cells were then harvested and lysed in 100 µL 1X SDS sample buffer (Boston Bio, #BP111NR) and boiled for 10 mins. Samples were run on NuPAGE 4 to 12% Bis-Tris Gels (Thermo Fisher) and transferred on nitrocellulose membrane with an iBlot2 (Thermo Fisher). Membranes were blocked in 5% non-fat milk in TBS Tween. Antibodies against phospho proteins were incubated in 5% BSA TBS tween. ECL signal was recorded on a ChemiDoc Biorad Imager. Data was analyzed with ImageLab (Biorad). Antibodies against STING (CST, #13647), pSTING (CST, #40818), ATG16L1 (CST, #8089S), mNeongreen (CST, #53061), LC3B (CST, #2775), Actin (abcam, #ab49900), and IL-1B (CST, #12703) were used for detecting indicated protein.

Monocyte Experiments

CD14+ monocytes were isolated from peripheral adult human blood using CD14 Microbeads (Miltenyi) as previously described *(3)*. CD14+ monocytes were cultured in RPMI (Gibco)

supplemented with 10% FBS (VWR), 1X GlutaMax (Thermo Fisher), and 1X Penicillin/Streptomycin (Corning). Monocytes were plated at 400,000 cells/well and stimulated with indicated priming reagents (2µg/ml Pam3CSK4, 1µg/ml R848) for 2h, and then stimulated with indicated stimuli (10µg/ml cGAMP, 1µM DiABZI, 10uM C53, 5µM MCC950) for 6h. 6.7µM nigericin was added in the last 2h of stimulation. All the stimuli were from Invivogen except C53 (Cayman). Stimulated cells were then centrifuged to separate cells and supernatant for PI staining, Legendplex assay and Immunoblot. Cytokine quantification was performed following standard Legendplex protocol using anti-IL1b beads (Biolegend, #740812). PI staining was performed in 120µl/well with a 1:200 PI (Biolegend) dilution. Cells for PI staining and beads for Legendplex were acquired on a Cytoflex LX (Beckman Coulter). Data was analyzed with FlowJo v10.7 (BD). IL-1β concentration was calculated using Graphpad Prism based on mean fluorescence intensity of a standard curve.

BlaER1 Experiments

BLaER1 were transduced with pXPR023-ATG16L1_g01 or pXPR023-ATG16L1_g02, selected with 2 µg/mL Puromycin and then trans-differentiated into monocytes as described in Gaidt et al 2018 (*34*). Briefly, 70,000 cells/well were plated in a 96 well flat bottom with 10 ng/mL IL-3 (Peprotech), 10 ng/mL, M-CSF (Biolegend), 100 nM β-Estradiol (Sigma) in RPMI for 5 days. Before stimulation, the media was replaced and cells were pre-stimulated with 2 µg/ml Pam3CSK4 (Invivogen) for 1 hour. Cells were then stimulated with the combination of drugs indicated in the figure legend for 5 hours. Supernatant was then collected and IL-1β was measured via Legendplex following manufacturer's instructions.

FACS Screen Analysis

Guide RNA abundances were extracted from FASTQ files using poolq 3.3.2 with fixed row and barcode policies. Resulting log-abundances were then subtracted between sorting bins from each experimental condition and analyzed using the Broad Genetic Perturbation Platform screen analysis tool (https://portals.broadinstitute.org/gpp/public/analysis-tools/crispr-gene-scoring) using a hypergeometric analysis.

Image Analysis

Analysis was performed using code adapted from *(4)*, and all analysis code is available on GitHub. For live-cell confocal imaging experiments with BJ1 cells expressing mRuby3 SEP, analysis was performed using z-stack maximum projection images. Cells were segmented by thresholding the Hoechst signal to identify nuclei and expanding the resulting regions to define cell areas using the seeded watershed method based on a defined threshold in the mRuby3 channel. All experiments used the same mRuby3 thresholds, while Hoechst thresholds were varied as appropriate to account for technical differences in staining levels. Segmented cells were tracked across timepoints and the ratio of maximum superecliptic pHluorin to median mRuby3 in the cytoplasm (defined as the difference between the cell and nuclear masks) was extracted in tracked cells. For pH calibration experiments, a single cell mask was defined at pH = 7.5 using the mRuby3 channel and applied to define cell regions at lower pH values that were subsequently measured. Linear regression (scikit-learn linear_model.LinearRegression) was used to separately determine correspondence between pH values and SEP max / mRuby3 median ratios for each genetically encoded reporter using measurements at pH values 6.5, 7, and 7.5 (the linear range for these reporters). For super-resolution Airyscan analysis, cells were manually masked based on background fluorescence in the STING miRFP680 channel. A single z-stack was selected for analysis and individual Golgi vesicles were segmented at each timepoint based on thresholding in the mRuby3 channel. SEP,

mRuby3, and STING miRFP680 intensities were separately extracted from each segmented vesicle. For each cell, initial SEP/mRuby3 ratios and STING intensity were normalized relative to baseline and segmented vesicles were defined as SEP/mRuby3 high if the ratio was >= 1.5-fold relative to the mean baseline ratio for each cell. For fixed cell analysis of LC3B lipidation and inflammasome induction experiments, nuclei were segmented by thresholding the DAPI signal and cells were segmented by expanding the resulting regions using the seeded watershed method. Cells were segmented based on background fluorescence in the STING or NLRP3 mNeonGreen channels for LC3B lipidation and inflammasome experiments, respectively. For the LC3B lipidation experiments, LC3B puncta were segmented based on a defined intensity threshold and area range in that channel. For the inflammasome experiments, around 50% of the cells were removed based on lack of expression of NLRP3 mNeonGreen or STING, as not all cells expressed both constructs. Across all experiments, the only analysis settings varied across replicates were the Hoechst threshold for nuclear segmentation of live-cell imaging experiments.

Figures S1-S4

Figure S1

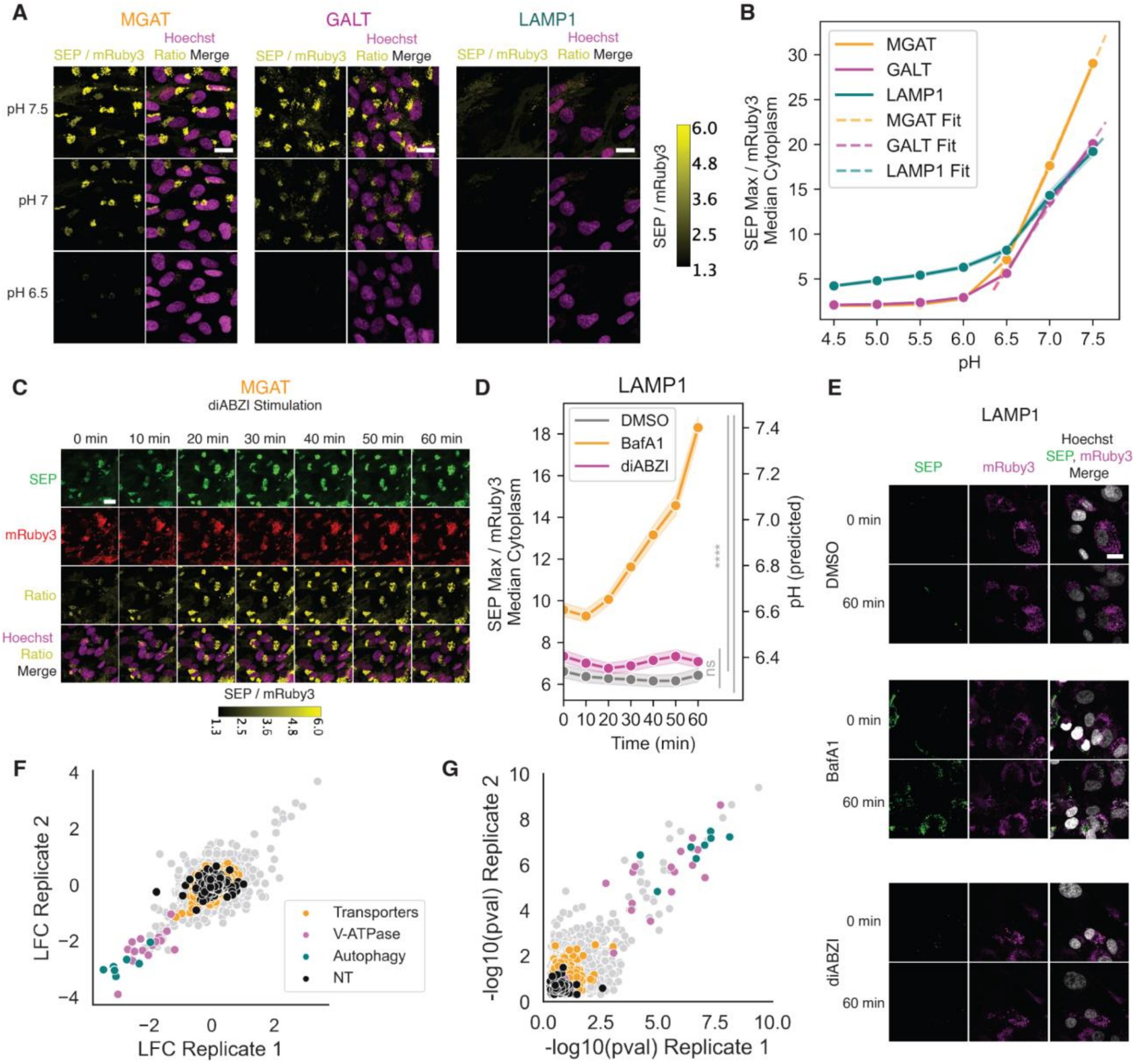
A
MGAT
GALT
LAMP1
SEP / mRuby3
Hoechst
Ratio Merge
pH 7.5
pH 7
pH 6.5
SEP / mRuby3
6.0
4.8
3.6
2.5
1.3
B
MGAT
GALT
LAMP1
MGAT Fit
GALT Fit
LAMP1 Fit
SEP Max / mRuby3 Median Cytoplasm
pH
C
MGAT
diABZI Stimulation
0 min
10 min
20 min
30 min
40 min
50 min
60 min
SEP
mRuby3
Ratio
Hoechst
Ratio
Merge
SEP / mRuby3
D
LAMP1
DMSO
BafA1
diABZI
SEP Max / mRuby3 Median Cytoplasm
pH (predicted)
Time (min)
****
ns
E
LAMP1
SEP
mRuby3
Hoechst
SEP, mRuby3
Merge
0 min
60 min
DMSO
BafA1
diABZI
F
LFC Replicate 2
LFC Replicate 1
Transporters
V-ATPase
Autophagy
NT
G
-log10(pval) Replicate 2
-log10(pval) Replicate 1

Figure S2

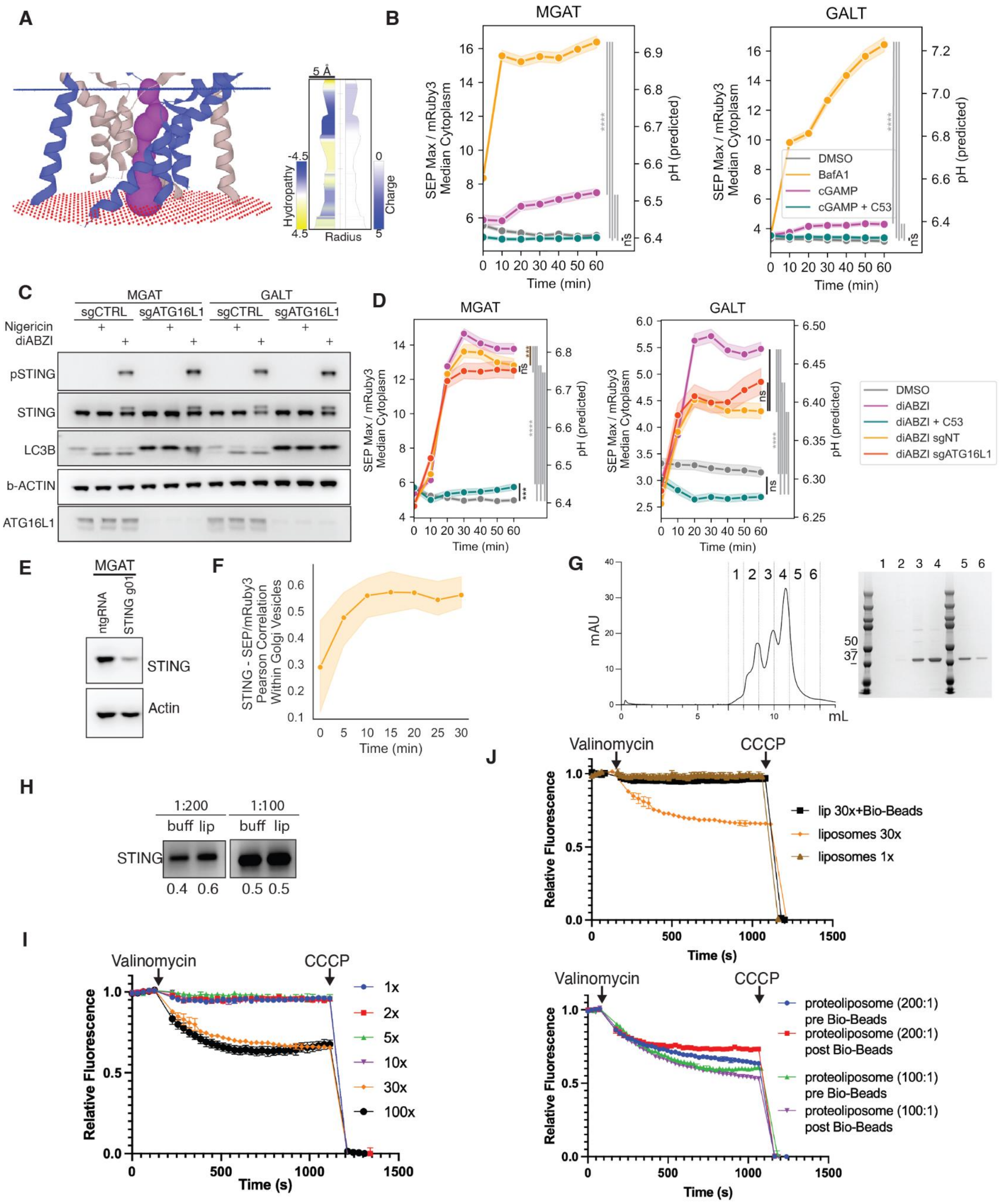

A
B
MGAT
GALT
SEP Max / mRuby3 Median Cytoplasm
pH (predicted)
Time (min)
DMSO
BafA1
cGAMP
cGAMP + C53
ns
Hydropathy
Charge
Radius
5 Å
C
sgCTRL
sgATG16L1
Nigericin
diABZI
pSTING
STING
LC3B
b-ACTIN
ATG16L1
D
diABZI
diABZI + C53
diABZI sgNT
diABZI sgATG16L1
E
ntgRNA
STING g01
Actin
F
STING - SEP/mRuby3 Pearson Correlation Within Golgi Vesicles
G
mAU
mL
H
1:200
1:100
buff
lip
0.4
0.6
0.5
0.5
I
Valinomycin
CCCP
Relative Fluorescence
Time (s)
1x
2x
5x
10x
30x
100x
J
lip 30x+Bio-Beads
liposomes 30x
liposomes 1x
proteoliposome (200:1) pre Bio-Beads
proteoliposome (200:1) post Bio-Beads
proteoliposome (100:1) pre Bio-Beads
proteoliposome (100:1) post Bio-Beads

Figure S3

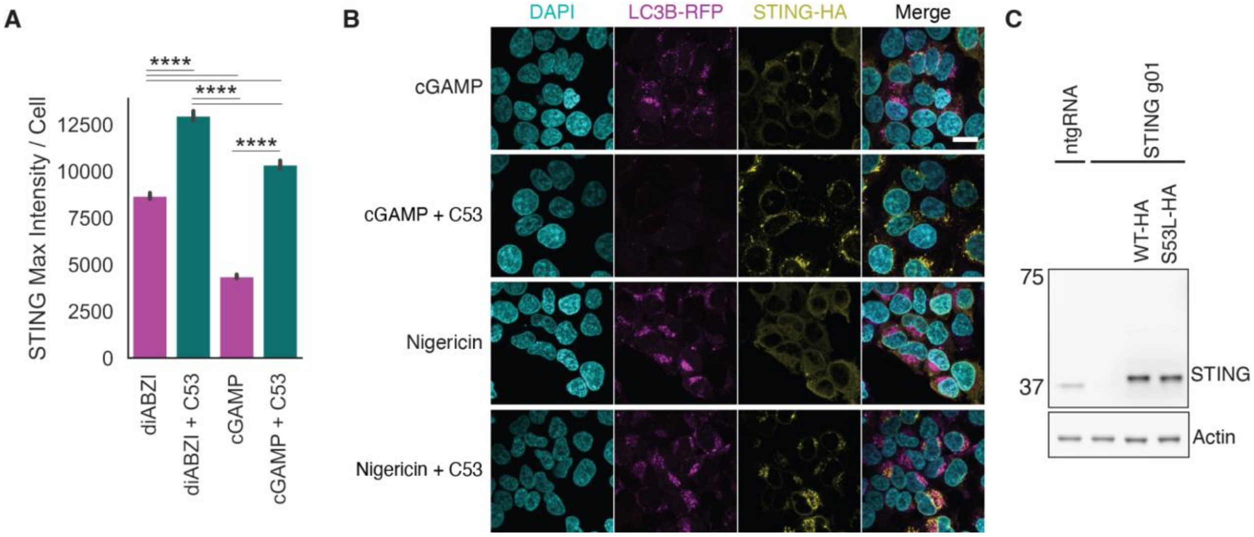
A
STING Max Intensity / Cell
0
2500
5000
7500
10000
12500
****
****
****
diABZI
diABZI + C53
cGAMP
cGAMP + C53
B
DAPI
LC3B-RFP
STING-HA
Merge
cGAMP
cGAMP + C53
Nigericin
Nigericin + C53
C
ntgRNA
STING g01
WT-HA
S53L-HA
75
37
STING
Actin

Figure S4

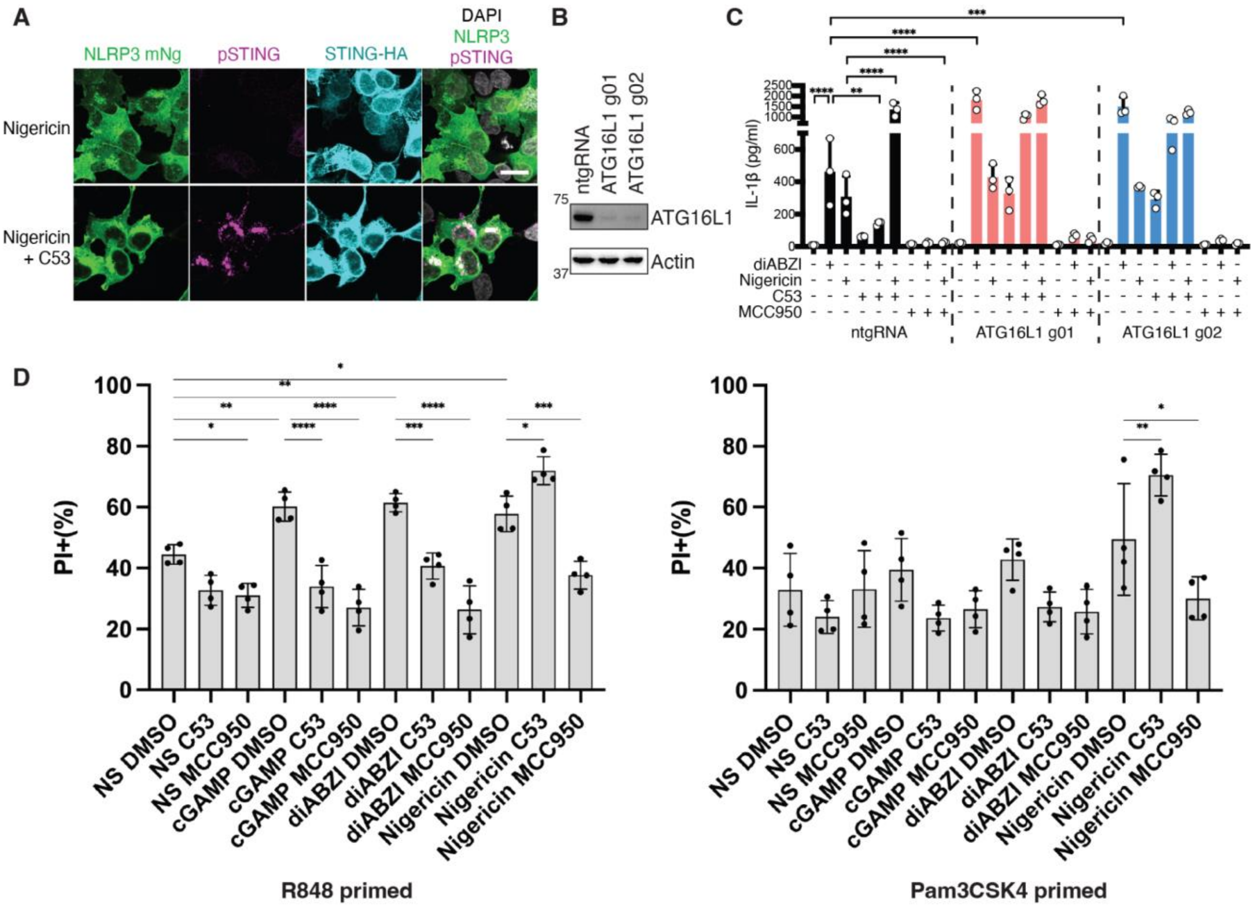
A
NLRP3 mNg
pSTING
STING-HA
DAPI
NLRP3
pSTING
Nigericin
Nigericin
+ C53
B
ntgRNA
ATG16L1 g01
ATG16L1 g02
75
37
ATG16L1
Actin
C
IL-1β (pg/ml)
diABZI
Nigericin
C53
MCC950
ntgRNA
ATG16L1 g01
ATG16L1 g02
D
PI+(%)
NS DMSO
NS C53
NS MCC950
cGAMP DMSO
cGAMP C53
cGAMP MCC950
diABZI DMSO
diABZI C53
diABZI MCC950
Nigericin DMSO
Nigericin C53
Nigericin MCC950
R848 primed
Pam3CSK4 primed

Figure S1. (**A**) Representative images of BJ1 cells expressing a ratiometric SEP and mRuby3 reporter localized to MGAT, GALT, or LAMP1 treated with 1 µM nigericin and 1 µM valinomycin and buffers with defined pH. Scale bar 20 µm. (**B**) Quantification of experiment in (A), data from three biological replicates combined, linear regression fits from pH 6.5 to 7.5 shown. Shaded region denotes SD. (C) Cropped image from Fig. 1a across all timepoints assayed showing each channel separately as well as the SEP to mRuby3 ratio and Hoechst merge. (**D**) Quantification of changes in LAMP1 reporter pH upon 1 µM diABZI or 1 µM BafA1 stimulation, data from three biological replicates combined. Shaded region denotes SD. One-way ANOVA followed by Tukey's HSD at 60 minute timepoint. **** indicates $p < .0001$, ns = $p > 0.05$ (**E**) Representative images of BJ1 cells in (D), scale bar 20 µm. (**F**) Replicate log2-fold-change correlations for genome-wide CRISPR screens. (**G**) Replicate -log10(FDR adjusted p value) correlations for genome-wide CRISPR screens.

Figure S2. (**A**) Radius, charge and hydropathy of the proposed channel area of chicken STING bound with cGAMP (PBD: 6NT7). (**B**) Quantification of pH in BJ1 SEP mRuby3 MGAT or GALT reporter cells from 0 to 60 minutes post 20 µg/mL cGAMP with 5 µg/ml digitonin or 1 µM BafA1 stimulation, with or without 10 µM C53; data from three biological replicates combined. Shaded region denotes SD. One-way ANOVA followed by Tukey's HSD at 60 minute timepoint. **** indicates $p < .0001$, ns $p > 0.05$. (**C**) Immunoblots of indicated proteins in BJ1 SEP mRuby3 MGAT or GALT reporter cells expressing Cas9 and nontargeting sgRNA (ntgRNA) or ATG16L1 targeting sgRNA (ATG16L1 g01). Cells were unstimulated, or stimulated with 2uM nigericin, or 1uM DiABZI for 1h. (**D**) Quantification of pH in BJ1 SEP mRuby3 MGAT or GALT reporter cells from 0 to 60 minutes post 1 µM diABZI stimulation with or without 10 µM C53 and a non-targeting sgRNA or an sgRNA targeting ATG16L1 (g01), data from three biological replicates combined. Shaded region denotes SD. One-way ANOVA followed by Tukey's HSD at 60 minute timepoint. **** indicates $p < .0001$, ns $p > 0.05$. (**E**) Immunoblots of indicated proteins in BJ1 SEP mRuby3 MGAT reporter cells expressing Cas9 and nontargeting sgRNA (ntgRNA) or STING targeting sgRNA (STING g01). (**F**) Quantification of super-resolution Airyscan images of BJ1 MGAT SEP mRuby3 STING KO cells overexpressing STING WT miRFP680 stimulated with 1 µM diABZI, representing four biological replicates and five individual cells. STING intensity and SEP/mRuby3 ratios were normalized to per-cell baseline median intensities and Pearson correlations for each cell between STING and SEP/mRuby3 in Golgi vesicles were calculated at each timepoint. Shaded region denotes SD. (**G**) Size exclusion chromatography of eluted STING protein (left) with coomassie staining (right) of each fractions. One representative experiment of n=3 experiments. Fractions 5 and 6 were combined for proteoliposome experiments. (**H**) Immunoblot of STING from buffer fraction (buff) or liposome fraction (lip). Samples are from the experiments that use 1:200 or 1:200 protein:lipid mass ratio. Liposomes were separated from the buffer through 30,000g spin for 30 minutes. (**I**) Detergent titration on empty liposomes. Liposomes were mixed with 1x to 100x the concentration of detergent used in the standard proteoliposome assays shown in the manuscript and then evaluated for their proton leakage. (**J**) Liposome and proteoliposome assay with or without Bio-Beads detergent removal. (Top) Proton leakage was measured after mixing liposomes with detergent at 1x, 30x concentration (relative to the amount used in our standard proteoliposomes assays) followed by none, or 3 rounds of Bio-Beads (lip 30x+Bio-Beads group) depletion of detergent. (Bottom) Proteoliposomes loaded with STING protein with lipid to protein mass ratio 200:1 or 100:1 were left untreated or went through 3 rounds of Bio-Beads detergent depletion, followed by the standard proteoliposome assay. One representative experiment of n=2 experiments for (**H)**, (**I)**, and (**J)**.

Figure S3. (**A**) STING translocation quantified as the per-cell maximum STING intensity for experiment in Figure 3A, representing three biological replicates combined. Error bars denote SD. One-way ANOVA followed by Tukey's HSD, **** indicates $p < .0001$. (**B**) Representative images of stably expressed RFP-LC3B and STING-HA in FIP200 KO 293T cells upon 20 μg/mL cGAMP with 5 μg/ml digitonin or 2 μM nigericin stimulation with or without 10 μM C53 co-treatment. Scale bar 20 μm. (**C**) Immunoblots of indicated proteins from BJ1 SEP mRuby3 MGAT cells transduced with Cas9 with non-targeting sgRNA (ntgRNA) or Cas9 with STING targeting sgRNA (STING g01) with indicated STING WT or STING S53L expression.

Figure S4. (**A**) Representative images of pSTING, STING, and NLRP3 in HEK293T cells expressing STING-HA and NLRP3-mNeonGreen and treated with 2 µM nigericin with or without 10 µM C53 for 1 hour. Scale bar 20 µm. (B) Immunoblots of indicated proteins in BLaER1 cells expressing Cas9 and nontargeting sgRNA (ntgRNA) or ATG16L1 targeting sgRNAs (ATG16L1 g01 and g02). (**C**) IL-1β quantification in supernatant of differentiated BLaER1 KO cells in (**B**) pre-treated for 1 hour with 2 µg/ml Pam3CSK4 and stimulated for 5 hours with the indicated combinations of drugs (1 µM diABZI, or 6.7 µM nigericin stimulation in the absence or presence of 10 µM C53 or the 5 µM NLRP3 inhibitor MCC950). Data was quantified using three combined biological replicates. Error bars denote SD. One-way ANOVA with post-hoc Tukey test on log-normalized data. ** $p<0.01$, *** $p<0.001$, **** $p<0.0001$. (**D**) PI staining of R848 (left) or Pam3CSK4 (right) primed monocytes upon NS (no stimulus), 10 µg/ml cGAMP, 1 µM diABZI, or 6.7 µM nigericin stimulation in the absence or presence of 10 µM C53 or the 5 µM NLRP3 inhibitor MCC950. Error bars denote SD. One-way ANOVA with post-hoc Tukey test between all DMSO-treated groups (DMSO plus NS, cGAMP, diABZI, and nigericin) and between matched DMSO-C53/MCC950 groups with the same stimulus (NS, cGAMP, diABZI, and nigericin). * $p<0.05$, ** $p<0.01$, *** $p<0.001$, **** $p<0.0001$, non-significant tests not shown. NS: no stimulation. Each data point represents one donor, total n = 4 donors.

Video S1. Live-cell confocal imaging of BJ1 cells expressing SEP and mRuby3 targeted to MGAT and stimulated with DMSO, 1 µM diABZI with or without 10 µM C53 for 60 minutes; cells imaged every 10 minutes. SEP/mRuby3 ratio in yellow, Hoechst in magenta. Scale bar 20 µm.

Video S2. Super-resolution Airyscan imaging of BJ1 cells expressing SEP and mRuby3 targeted to MGAT and STING-miRFP680 and stimulated with 1 µM diABZI for 30 minutes; cells imaged every 5 minutes. SEP in orange, mRuby3 in blue, STING-miRFP680 in purple. Scale bar 5 µm.

Table S2. List of sgRNAs.

sgRNA Name	sgRNA Sequence	Vector
ntgRNA	GTATTACTGATATTGGTGGG	pXPR_BRD023
STING_g01	CATTACAACAACCTGCTACG	pXPR_BRD023
ATG16L1_g01	GCTGCAGAGACAGGCGTTCG	pXPR_BRD023
ATG16L1_g02	AAAAGCATGACGTACCAAAC	pXPR_BRD023

Table S3. List of Zenodo datasets.

**Dataset Name**	**Link**
GitHub Repository	https://doi.org/10.5281/zenodo.7938732
Live-cell MGAT, GALT, and LAMP1 pH Measurements Upon BafA1 and diABZI treatment	https://doi.org/10.5281/zenodo.7761368
Live-cell pH Calibration Meaurements	https://doi.org/10.5281/zenodo.7761419
Live-cell MGAT pH Measurement upon STING agonist treatment with or without C53	https://doi.org/10.5281/zenodo.7761405
Live-cell GALT pH Measurement upon STING agonist treatment with or without C53	https://doi.org/10.5281/zenodo.7761409
Live-cell MGAT Super-Resolution Experiment	https://doi.org/10.5281/zenodo.7761740
Live-cell MGAT STING WT or S53L pH Measurement upon STING agonist treatment with or without C53	https://doi.org/10.5281/zenodo.7783355
HEK293T Autophagy Experiment	https://doi.org/10.5281/zenodo.7761451
HEK293T Inflammasome Experiment	https://doi.org/10.5281/zenodo.7761453

Table S4. List of plasmids.

Plasmid Name	Addgene Link
RFP-LC3B	https://www.addgene.org/200943/
NLRP3-mNeonGreen	https://www.addgene.org/200942/
STING-miRFP680	https://www.addgene.org/200940/
MGAT-SEP-mRuby3	https://www.addgene.org/200937/
GALT-SEP-mRuby3	https://www.addgene.org/200938/
LAMP1-SEP-mRuby3	https://www.addgene.org/200939/

References and Notes

1. D. L. Burdette, K. M. Monroe, K. Sotelo-Troha, J. S. Iwig, B. Eckert, M. Hyodo, Y. Hayakawa, R. E. Vance, STING is a direct innate immune sensor of cyclic di-GMP. *Nature* **478**, 515–518 (2011).

2. L. Sun, J. Wu, F. Du, X. Chen, Z. J. Chen, Cyclic GMP-AMP synthase is a cytosolic DNA sensor that activates the type I interferon pathway. *Science* **339**, 786–791 (2013).

3. J. Wu, L. Sun, X. Chen, F. Du, H. Shi, C. Chen, Z. J. Chen, Cyclic GMP-AMP is an endogenous second messenger in innate immune signaling by cytosolic DNA. *Science* **339**, 826–830 (2013).

4. H. Ishikawa, G. N. Barber, STING is an endoplasmic reticulum adaptor that facilitates innate immune signalling. *Nature* **455**, 674–678 (2008).

5. X. Gui, H. Yang, T. Li, X. Tan, P. Shi, M. Li, F. Du, Z. J. Chen, Autophagy induction via STING trafficking is a primordial function of the cGAS pathway. *Nature* **567**, 262–266 (2019).

6. M. M. Gaidt, T. S. Ebert, D. Chauhan, K. Ramshorn, F. Pinci, S. Zuber, F. O'Duill, J. L. Schmid-Burgk, F. Hoss, R. Buhmann, G. Wittmann, E. Latz, M. Subklewe, V. Hornung, The DNA Inflammasome in Human Myeloid Cells Is Initiated by a STING-Cell Death Program Upstream of NLRP3. *Cell* **171**, 1110–1124.e18 (2017).

7. C. Zhang, G. Shang, X. Gui, X. Zhang, X.-C. Bai, Z. J. Chen, Structural basis of STING binding with and phosphorylation by TBK1. *Nature* **567**, 394–398 (2019).

8. B. Zhao, F. Du, P. Xu, C. Shu, B. Sankaran, S. L. Bell, M. Liu, Y. Lei, X. Gao, X. Fu, F. Zhu, Y. Liu, A. Laganowsky, X. Zheng, J.-Y. Ji, A. P. West, R. O. Watson, P. Li, A conserved PLPLRT/SD motif of STING mediates the recruitment and activation of TBK1. *Nature* **569**, 718–722 (2019).

9. Y. Xu, S. Cheng, H. Zeng, P. Zhou, Y. Ma, L. Li, X. Liu, F. Shao, J. Ding, ARF GTPases activate Salmonella effector SopF to ADP-ribosylate host V-ATPase and inhibit endomembrane damage-induced autophagy. *Nat. Struct. Mol. Biol.* **29**, 67–77 (2022).

10. T. D. Fischer, C. Wang, B. S. Padman, M. Lazarou, R. J. Youle, STING induces LC3B lipidation onto single-membrane vesicles via the V-ATPase and ATG16L1-WD40 domain. *J. Cell Biol.* **219**, e202009128 (2020).

11. K. M. Hooper, E. Jacquin, T. Li, J. M. Goodwin, J. H. Brumell, J. Durgan, O. Florey, V-ATPase is a universal regulator of LC3-associated phagocytosis and non-canonical autophagy. *J. Cell Biol.* **221**, e202105112 (2022).

12. Y. Xu, P. Zhou, S. Cheng, Q. Lu, K. Nowak, A.-K. Hopp, L. Li, X. Shi, Z. Zhou, W. Gao, D. Li, H. He, X. Liu, J. Ding, M. O. Hottiger, F. Shao, A Bacterial Effector Reveals the V-ATPase-ATG16L1 Axis that Initiates Xenophagy. *Cell* **178**, 552–566.e20 (2019).

13. R. Ulferts, E. Marcassa, L. Timimi, L. C. Lee, A. Daley, B. Montaner, S. D. Turner, O. Florey, J. K. Baillie, R. Beale, Subtractive CRISPR screen identifies the ATG16L1/vacuolar ATPase axis as required for non-canonical LC3 lipidation. *Cell Rep.* **37**, 109899 (2021).

14. G. Miesenböck, D. A. De Angelis, J. E. Rothman, Visualizing secretion and synaptic transmission with pH-sensitive green fluorescent proteins. *Nature* **394**, 192–195 (1998).

15. S. Sankaranarayanan, D. De Angelis, J. E. Rothman, T. A. Ryan, The use of pHluorins for optical measurements of presynaptic activity. *Biophys. J.* **79**, 2199–2208 (2000).

16. P. T. A. Linders, M. Ioannidis, M. Ter Beest, G. van den Bogaart, Fluorescence Lifetime Imaging of pH along the Secretory Pathway. *ACS Chem. Biol.* **17**, 240–251 (2022).

17. K. R. Sanson, R. E. Hanna, M. Hegde, K. F. Donovan, C. Strand, M. E. Sullender, E. W. Vaimberg, A. Goodale, D. E. Root, F. Piccioni, J. G. Doench, Optimized libraries for CRISPR-Cas9 genetic screens with multiple modalities. *Nat. Commun.* **9**, 5416 (2018).

18. K. E. Eng, M. D. Panas, G. B. Karlsson Hedestam, G. M. McInerney, A novel quantitative flow cytometry-based assay for autophagy. *Autophagy* **6**, 634–641 (2010).

19. D. Liu, H. Wu, C. Wang, Y. Li, H. Tian, S. Siraj, S. A. Sehgal, X. Wang, J. Wang, Y. Shang, Z. Jiang, L. Liu, Q. Chen, STING directly activates autophagy to tune the innate immune response. *Cell Death Differ.* **26**, 1735–1749 (2019).

20. G. Shang, C. Zhang, Z. J. Chen, X.-C. Bai, X. Zhang, Cryo-EM structures of STING reveal its mechanism of activation by cyclic GMP-AMP. *Nature* **567**, 389–393 (2019).

21. M. Gentili, B. Liu, M. Papanastasiou, D. Dele-Oni, M. A. Schwartz, R. J. Carlson, A. M. Al'Khafaji, K. Krug, A. Brown, J. G. Doench, S. A. Carr, N. Hacohen, ESCRT-dependent STING degradation inhibits steady-state and cGAMP-induced signalling. *Nat. Commun.* **14**, 611 (2023).

22. L. Pravda, D. Sehnal, D. Toušek, V. Navrátilová, V. Bazgier, K. Berka, R. Svobodová Vareková, J. Koca, M. Otyepka, MOLEonline: A web-based tool for analyzing channels, tunnels and pores (2018 update). *Nucleic Acids Res.* **46**, W368–W373 (2018).

23. D. Lu, G. Shang, J. Li, Y. Lu, X.-C. Bai, X. Zhang, Activation of STING by targeting a pocket in the transmembrane domain. *Nature* **604**, 557–562 (2022).

24. M.-F. Tsai, C. Miller, Substrate selectivity in arginine-dependent acid resistance in enteric bacteria. *Proc. Natl. Acad. Sci. U.S.A.* **110**, 5893–5897 (2013).

25. H.-H. Shen, T. Lithgow, L. Martin, Reconstitution of membrane proteins into model membranes: Seeking better ways to retain protein activities. *Int. J. Mol. Sci.* **14**, 1589–1607 (2013).

26. M. Dezi, A. Di Cicco, P. Bassereau, D. Lévy, Detergent-mediated incorporation of transmembrane proteins in giant unilamellar vesicles with controlled physiological contents. *Proc. Natl. Acad. Sci. U.S.A.* **110**, 7276–7281 (2013).

27. E. Jacquin, S. Leclerc-Mercier, C. Judon, E. Blanchard, S. Fraitag, O. Florey, Pharmacological modulators of autophagy activate a parallel noncanonical pathway driving unconventional LC3 lipidation. *Autophagy* **13**, 854–867 (2017).

28. T. Ichinohe, I. K. Pang, A. Iwasaki, Influenza virus activates inflammasomes via its intracellular M2 ion channel. *Nat. Immunol.* **11**, 404–410 (2010).

29. J. Chen, Z. J. Chen, PtdIns4P on dispersed trans-Golgi network mediates NLRP3 inflammasome activation. *Nature* **564**, 71–76 (2018).

30. B. Liu, R. J. Carlson, liucarlson2023/STINGChannel: Human STING is a proton channel, Version v02, Zenodo (2023); https://doi.org/10.5281/zenodo.7938732.

31. J.-L. Rigaud, D. Lévy, Reconstitution of membrane proteins into liposomes. *Methods Enzymol.* **372**, 65–86 (2003).

32. S. Han, S. Peng, J. Vance, K. Tran, N. Do, N. Bui, Z. Gui, S. Wang, Structural dynamics determine voltage and pH gating in human voltage-gated proton channel. *eLife* **11**, e73093 (2022).

33. S. Lee, H. Zheng, L. Shi, Q.-X. Jiang, Reconstitution of a Kv channel into lipid membranes for structural and functional studies. *J. Vis. Exp.* **77**, e50436 (2013).

34. M. M. Gaidt, F. Rapino, T. Graf, V. Hornung, Modeling Primary Human Monocytes with the Trans-Differentiation Cell Line BLaER1. *Methods Mol. Biol.* **1714**, 57–66 (2018).
